# Sigma-2 receptor agonist derivatives of 1-Cyclohexyl-4-[3-(5-methoxy-1,2,3,4-tetrahydronaphthalen-1-yl)propyl]piperazine (PB28) induce cell death via mitochondrial superoxide production and caspase activation in pancreatic cancer

**DOI:** 10.1186/s12885-016-3040-4

**Published:** 2017-01-13

**Authors:** Maria Laura Pati, John R. Hornick, Mauro Niso, Francesco Berardi, Dirk Spitzer, Carmen Abate, William Hawkins

**Affiliations:** 1 Dipartimento di Farmacia-Scienze del Farmaco, Università degli Studi di Bari ALDO MORO, Via Orabona 4, I-70125 Bari, Italy; 2 Department of Surgery, Division of Hepatobiliary, Pancreatic, and Gastrointestinal Surgery, Washington University School of Medicine, St. Louis, MO USA

**Keywords:** Sigma-2 receptor, Pancreatic cancer, Mitochondrial superoxide, Caspase-3 activity, Reactive oxygen species

## Abstract

**Background:**

Despite considerable efforts by scientific research, pancreatic cancer is the fourth leading cause of cancer related mortalities. Sigma-2 receptors, which are overexpressed in several tumors, represent promising targets for triggering selective pancreatic cancer cells death.

**Methods:**

We selected five differently structured high-affinity sigma-2 ligands (PB28, PB183, PB221, F281 and PB282) to study how they affect the viability of diverse pancreatic cancer cells (human cell lines BxPC3, AsPC1, Mia PaCa-2, and Panc1 and mouse Panc-02, KCKO and KP-02) and how this is reflected in vivo in a tumor model.

**Results:**

Important cytotoxicity was shown by the compounds in the aggressive Panc02 cells, where cytotoxic activity was caspase-3 independent for four of the five compounds. However, both cytotoxicity and caspase-3 activation involved generation of Reactive Oxygen Species (ROS), which could be partially reverted by the lipid antioxidant α-tocopherol, but not by the hydrophilic N-acetylcysteine (NAC) indicating crucial differences in the intracellular sites exposed to oxidative stress induced by sigma-2 receptor ligands. Importantly, all the compounds strongly increased the production of mitochondrial superoxide radicals except for PB282. Despite a poor match between in vitro and the in vivo efficacy, daily treatment of C57BL/6 mice bearing Panc02 tumors resulted in promising effects with PB28 and PB282 which were similar compared to the current standard-of-care chemotherapeutic gemcitabine without showing signs of systemic toxicities.

**Conclusions:**

Overall, this study identified differential sensitivities of pancreatic cancer cells to structurally diverse sigma-2 receptor ligands. Of note, we identified the mitochondrial superoxide pathway as a previously unrecognized sigma-2 receptor-activated process, which encourages further studies on sigma-2 ligand-mediated cancer cell death for the targeted treatment of pancreatic tumors.

## Background

Despite considerable efforts in the field of oncology, cancer remains one of the major public health problem worldwide. Pancreatic cancer is the fourth leading cause of cancer related mortalities with an overall five-year survival rate of only 7% [1]. Treatment with standard therapies offers just some discreet prolongation of survival [2], while preserving the best possible quality of life [3]. The prognosis for pancreatic cancer remains poor due to a high rate of local recurring event and metastatic disease [2]. Therefore, development of alternative strategies for tumor treatment is strongly needed. Novel compounds that exert anticancer effect acting through diverse tumor-specific molecular targets are under study, and sigma receptors ligands are among them. Sigma receptors subtypes, namely sigma-1 and sigma-2, are endocellular proteins highly expressed in many tumors [4]. The sigma-1 receptor has been shown to exert neuroprotective and neuroregulatiory functions is linked to CNS pathologies such as schizophrenia, depression, Parkinson’s and Alzheimer’s diseases [5–10].

**Fig. 1:**
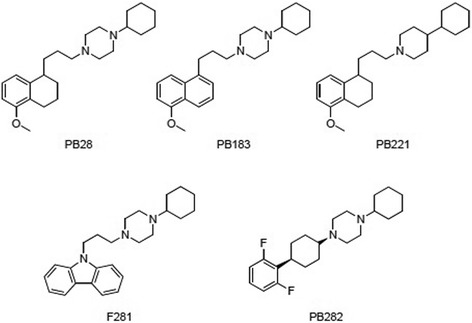
Sigma-2 receptor ligands PB28 and its analogues PB183, PB221, F281, PB282

The sigma-2 subtype, has not yet been cloned, and although its identification remains controversial [11–14], increasing interest in this protein is due to its overexpression and activity in a number of human tumors. Several sigma-2 ligands cause tumor selective cytotoxicity and apoptosis, although the mechanism of cell death induction is currently poorly understood but has been shown to involve caspase-dependent and -independent apoptosis, generation of reactive oxygen species (ROS), and autophagy [15–20]. On the basis of previous studies that showed certain sigma-2 agonists to be quite effective in preclinical tumor models of pancreatic cancer [21–24], we selected five sigma-2 ligands (Fig. 1, PB28, PB183, PB221, F281 and PB282). While PB28 is a well known sigma-2 agonist, the other compounds are variants of PB28 with different hydrophobic or basic moieties which were previously shown to bind sigma-2 receptors with high affinity (Table 1) [25–28]. In our current study, we systematically tested these novel compounds for relative effectiveness and relative toxicity in pancreatic cancer in vitro and in vivo. While various sigma-2 receptor ligands were capable of inducing apoptosis in tumor cells, the activation of caspase-3 and upstream signaling events leading to cell death appeared to depend on a particular combination of ligand and a given cell type.

**Table 1:** Sigma-2 binding affinity and pancreatic cancer cell line viability, following sigma-2 receptor ligand treatment (24 h)

Cmpd	Affinity (*K*_i_, nM)	Activity (EC_50_, μM)						
	σ_2_	Panc02	KP02	KCKO	MIAPaCa-2	BxPC3	AsPC-1	Panc-1
PB28^a^	0.68	43	>100	87	51	>100	>100	>100
PB221^a^	18.8	37	>100	19	50	100	>100	>100
PB183^b^	9.24	83	97	51	49	100	>100	>100
F281^c^	12.6	37	49	62	29	42	98	>100
PB282^d^	14.1	>100	>100	90	51	>100	>100	>100

^a^Sigma-2 binding data are from Berardi, et al. 2009 [26]; ^b^Sigma-2 binding data are from Berardi, et al. 2004 [25]; ^c^Sigma-2 binding data are from Ferorelli et al., 2007 [27]; ^d^Sigma-2 binding data are from Abate, et al., 2011 [28]. Pancreas cancer cell lines were treated with escalating doses of the sigma-2 ligands PB28, PB221, PB183, F281, PB282 or DMSO vehicle for 24 h and viability relative to vehicle determined by MTT Assay

Herein we evaluated variants of PB28 in preclinical models for efficacy in addition to define a mechanism of action (mitochondrial superoxide production) that has not been previously linked to a defined receptor mediated process. Better understanding of the sensitivity of cancers to some death pathways will help in tailoring the sigma-2 receptor ligand treatment choice.

## Methods

### Cell culture

Human pancreas cancer cell lines BxPC3, AsPC1, Mia PaCa-2, and Panc1 were obtained from American Type Culture Collection (ATCC, Bethesda, MD; catalog number: CRL-1687, CRL-1682, CRL-1420, and CRL-1469 respectively). Murine pancreas adenocarcinoma Panc02 was a gift from Bryan Clary (Duke University). The mouse KCKO cell line isolated from a spontaneously developing pancreatic cancer overexpressing human MUC1 [29, 30] was kindly provided by Dr. Pinku Mukherjee (University of North Carolina, Charlotte, NC). The mouse KP-2 line was derived from pancreatic cancer tumor tissue obtained from p48-CRE/LSL-Kras^G12D^/p53^flox/+^ mice (backcrossed C57BL/6, *n* = 6). AsPC-1, BxPC-3 and Panc02 cells were cultured in RPMI-1940 medium with 10% fetal bovine serum (FBS). MIA PaCa-2 cells were cultured in Dulbecco’s Modified Eagle’s Medium (DMEM) with 10% FBS and 2.5% horse serum. PANC-1 cells were cultured in DMEM with 10% FBS. KCKO cells were cultured in RPMI-1940 medium with 10% FBS, 1% sodium pyruvate, 1% HEPES buffer, and 1% L-glutamine. KP-2 cells were cultured in 1:1 mixture of DMEM and Ham’s F-12 Nutrient Mixture with 10% FBS. Penicillin (100 mg/mL) and streptomycin (100 mg/mL) were added to all media; cells were maintained in a humidified incubator at 37 °C with 5% CO_2_.

### Compounds

Sigma-2 receptor ligands PB28 [1-Cyclohexyl-4-[3-(5-methoxy-1,2,3,4-tetrahydronaphthalen-1-yl)propyl] piperazine], PB183 [1-Cyclohexyl-4-[3-(5-methoxynaphthalen-1-yl)propyl]- piperazine], PB221 [4-Cyclohexyl-1-[3-(5-methoxy-1,2,3,4-tetrahydronaphthalen- 1-yl-propyl)]piperidine], F281 [1-Cyclohexyl-4-[3-(9H-carbazol-9-yl)propyl]piperazine], and PB282 [*cis*-1-Cyclohexyl-4-[(2,6-difluorophenyl)cyclohexyl]piperazine] were synthesized in our laboratories according to published methods [25–28]. Gemcitabine and caspase-3 inhibitor Z-DEVD-FMK were purchased from Tocris Bioscience, α-tocopherol and *N*-Acetyl-L-cysteine from Sigma-Aldrich (St. Louis, MO). Compounds were dissolved in DMSO with final concentrations less than 0.3%.

### Cell viability and ROS interference

Determination of cell growth was performed using the MTT assay at 24 h [31, 32]. On day 1, pancreatic cancer cell lines were plated at a density of 2 × 10^4^ cells/well in black clear-bottom 96-well plates for 24 h prior to treatment in a volume of 100 μL. On day 2, the cells were treated for 24 h with the various drugs concentration (1 μM-100 μM). Untreated cells served as a control. The various drug-solvents (EtOH, DMSO) were added in each control to evaluate a possible solvent cytotoxicity. After the established incubation time with drugs (24 h), MTT (0.5 mg/ml) was added to each well, and after 3–4 h incubation at 37 °C, the supernatant was removed. The formazan crystals were solubilized using 100 μl of DMSO/EtOH (1:1) and the absorbance values at 570 and 630 nm were determined on the multi-mode microplate reader (BioTek instruments, Winooski, VT). Different drug concentrations were assayed in triplicates.

The interference of ROS in cell viability was indirectly determined by MTT assay reported above at 24 h. On day 1, Panc02 cells were plated at a density of 2 × 10^4^ cells/well in opaque 96-well, clear-bottom plates 24 h prior to treatment. On day 2, cells were treated with the drugs (50 μM) in the presence or absence of α-tocopherol (100 μM) or *N*-Acetyl-L-cysteine (100 μM). After incubation (24 h) with drugs, MTT assay was performed as above.

### Detection of caspase-3 activity in vitro

Caspase-3 activity was measured in Panc02 cell lines with a Caspase-Glo® Assay Systems (Promega) according to protocol in which the reagent contain luminogenic caspase substrates that cleaved by activated caspase. Cells were seeded at a density of 1 × 10^4^ in black 96-well, clear bottom plates for 24 h before treatment with 200 μM of compounds in presence or absence of α-tocopherol (100 μM) or Z-DEVD-FMK (1μM) for 24 h after treatment. The contents were then mixed using plate shaker for 30 s, and incubated at room temperature for 90 min. Luminescence signal was measured using multi-mode microplate reader (BioTek). Assay was performed in triplicates, and caspase activity was plotted compared cells treated with DMSO as a control.

### Detection of mitochondrial superoxide by flow cytometry and ROS interference

MitoSOX™ Red reagent is a novel fluorogenic dye specifically targeted to mitochondria in live cells. Oxidation of MitoSOX™ Red reagent by superoxide produces red fluorescence. Mitochondrial superoxide is generated as a byproduct of oxidative phosphorylation. In an otherwise tightly coupled electron transport chain, approximately 1–3% of mitochondrial oxygen consumed is incompletely reduced; those “leaky” electrons can quickly interact with molecular oxygen to form superoxide anion, the predominant reactive oxygen species (ROS) in mitochondria [33–36]. MitoSOX™ Red reagent is readily oxidized by superoxide but not by other ROS- or reactive nitrogen species (RNS)–generating systems. Panc02, AsPC-1, KP-2 or BxPC3 cells were seeded into 12-well plates 24 h before treatment with sigma-2 ligands (50 μM) for 2 h at 37°C in presence or absence of α-tocopherol (100 μM) followed by staining with MitoSOX™ Red (5 μM). Two hours after red dye addition, the cells were washed twice with PBS and harvested with trypsin/EDTA buffer. The cells were washed twice with PBS before analysis with FACSCalibur (BD Bioscience, San Jose, CA). The oxidation product of MitoSOX™ Red by mitochondrial superoxide fluoresces with an emission maximus of 580 nm and was detected in the FL3 channel. Experiment was performed in triplicates.

### In vivo assessment of tumor growth and survival

Animal studies were performed according to the animal studies protocol (20130073) approved by the Washington University Institutional Animal Care Facility. In this pre-clinical model, we utilized the Panc02 cell line [37], which is weakly immunogenic in C57BL/6 mice and aggressive with a subcutaneous inoculum of 1 × 10^5^ cells being lethal within 6–8 weeks. In vivo studies with mice were performed to compare the effect of sigma-2 ligands with gemcitabine. Mice treated with vehicle alone (a mixture of 5% DMSO, 5% EtOH, and 10% Cremophor in H_2_O) served as the control cohort. Female C57BL/6 mice (10 weeks old, National Cancer Institute Laboratories) were injected in the right flank with 200 μL of a single-cell suspension of Panc02 cells in non-supplemented RPMI medium (1 × 10^6^ cells per mouse). Treatment began when the mean tumor diameter was ~ 5 mm. Mice received daily intraperitoneal (i.p.) injections of the sigma-2 ligands PB28 (1.07 mg), PB221 (1.1 mg), PB183 (1.06 mg), F281 (1.05 mg), PB282 (1.09 mg), or gemcitabine (3 mg, once weekly) in 100 μL vehicle or vehicle alone (control) for 2 weeks. Tumors were measured three times weekly in two dimensions with a digital caliper, and tumor volumes were calculated by the standard formula of Tumor Volume = Length x Width^2^ × 0.5. All mice were euthanized when tumors reached a diameter of 15 mm or had ulcerated.

### Toxicity evaluation on mice not bearing tumor

Female C57BL/6 mice (8 weeks old, National Cancer Institute Laboratories) received daily i.p. injections of the sigma-2 ligands PB28 (1.07 mg), PB221 (1.1 mg), PB183 (1.06 mg), F281 (1.05 mg), PB282 (1.09 mg), in 100 μL vehicle or vehicle alone (control) for 1 week. Mice from each treatment cohort were assessed for pathologic evaluation (Digestive Diseases Research Core Center at our institution). Blood was collected for complete blood count (CBC) and biochemical analysis (AST, ALT, BUN, total protein, glucose and Cr). Organs were examined grossly and histologically.

### Statistical analysis

Statistical analyses and data plotting were performed using GraphPad Prism software version 6.03 (San Diego, CA). Results were expressed as mean ± standard error of the mean of at least 3 biological replicates. EC_50_ values were calculated by curve fitting normalized viability versus drug concentration. Differences in viability, caspase-3 activity, and tumor volume were analyzed using two-way ANOVA to identify differences and confirmed with paired two tailed t-tests. Mann-Whitney test was used to compare the difference in CBC and biochemistry analyses. Kaplan-Meier survival analyses were used to assess differences between treatment groups and were compared using a log-rank test. A *p-*value < 0.05 was considered significant for all analyses.

## Results

### Efficacy of PB28 variants against pancreatic cancer cell lines in vitro

We assessed the cell killing activity of our drugs on a panel of human (MIAPaCa-2, BxPC3, AsPC-1 and Panc-1) and mouse (Panc02, KP-2 and KCKO) pancreatic cancer cell lines in vitro. The majority of the five compounds tested (Fig. 1, PB28, PB221, PB183, F281 and PB282) reduced the viability in Panc02 (except for PB282), KCKO and MIAPaCa-2 (Table 1). However, some cell lines (AsPC1 and Panc-1) were resistant to all the compounds we tested as demonstrated by EC_50_ > 100 μM. Two cell lines (KP02 and BxPC3) were particularly sensitive to F281 as demonstrated by intermediate anti-proliferative activity (Table 1 EC50 ~ 45 μM). Notable differences were recorded in the effect of the same compounds among cells, such as F281 in Panc02, AsPC-1 and Panc-1 cells (EC_50_ values = 37 μM, 98 μM or 100 μM, respectively). Where active, compounds PB221 and F281 displayed the most potent anti-proliferative activity with F281 displaying a certain effect also in the BxPC3 cells. These results, while suggesting differential sensitivities of cells to sigma-2 receptor ligands, support their potential as therapeutics for the treatment of pancreatic cancer.

### Sigma-2 ligands induce caspase-3 activation

**Fig. 2:**
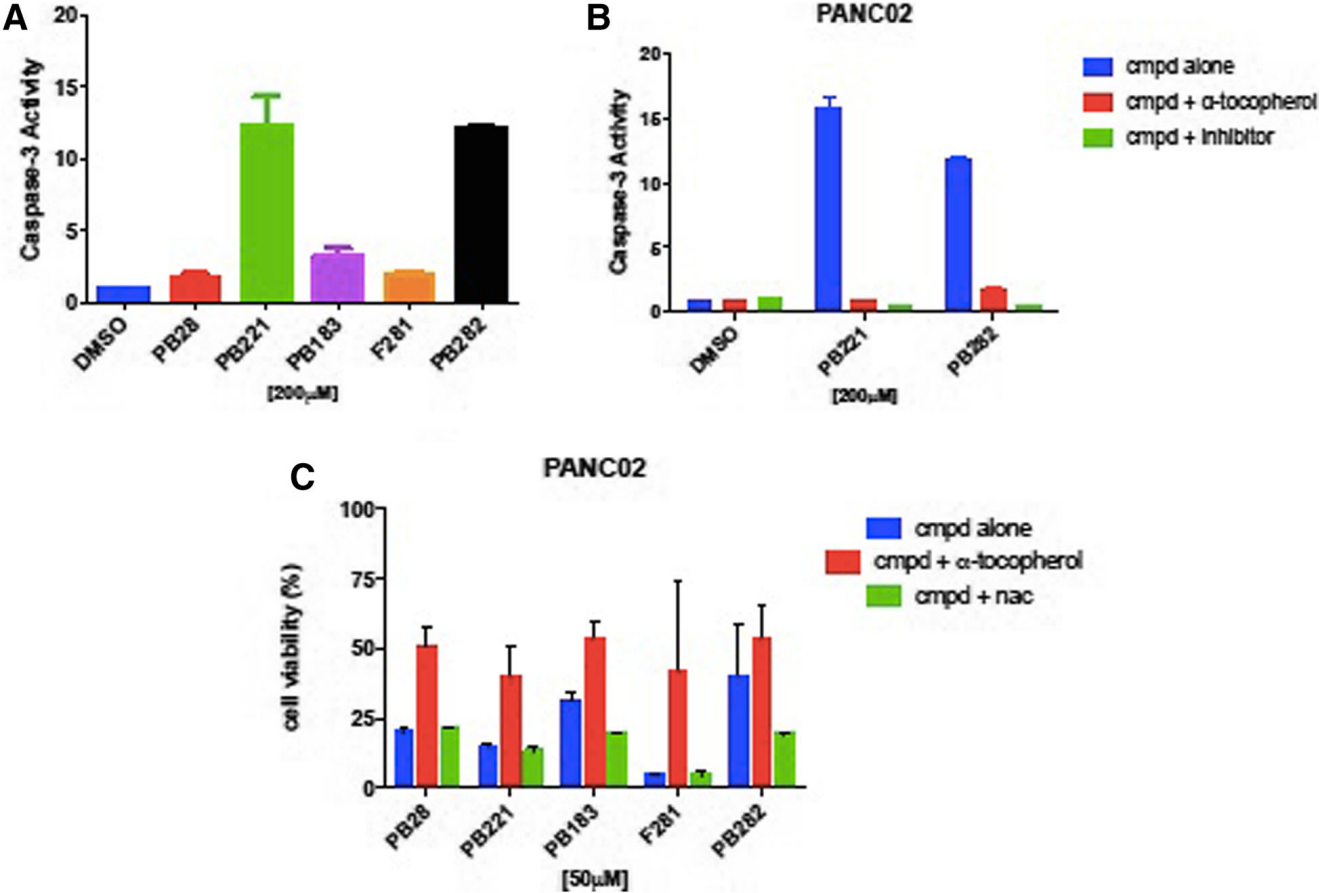
Sigma-2 ligands induce caspase-3 activation and involve Reactive Oxygen Species (ROS) in Panc02 cell lines (**a**) Caspase-3 activation was measured by Caspase-GloR Assay in Panc02 cells treated with 200 μM of different compounds for 5 h and expressed relative to vehicle. Cells treated with PB221 and PB282 had significant increase in caspase-3 *p* < 0.0001. **b** Panc02 cells were pre-treated with caspase-3 inhibitor Z-DEVD-FMK (1 μM) or DMSO vehicle for one hour prior to exposure to sigma-2 ligands PB221, PB282 (200 μM) or DMSO vehicle for 5 h. The caspase-3 specific inhibitor Z-DEVD-FMK and lipophilic antioxidant α-tocopherol abrogates caspase-3 dependent cleavage *p* < 0.0001. **c** Indirect measurement of ROS involvement following 24 h treatment with 50 μM PB28, PB221, PB183, F281 or PB282 in the presence of 100 μM lipophilic antioxidant α-tocopherol or hydrophilic antioxidant N-acetyl-L-cysteine (NAC), *p* = 0.002

Sigma-2 ligands activated mechanisms are cell and ligand specific and may cause both caspase-3 dependent and independent apoptosis [15, 16, 22]. In order to test the effects of our structurally diverse sigma-2 ligands regarding the mechanism of cell death induction, activation of caspase-3 was assessed initially. To compare caspase-3 activity induced by PB28, PB221, PB183, F281 and PB282, Panc02 mouse adenocarcinoma cells were treated with sigma-2 ligands (200 μM) for 5 h and assayed for cleavage of the proluminescent caspase-3 substrate and subsequent generation of a glow-type luminescent signal. PB221 and PB282 similarly induced a strong activation of caspase-3, increasing caspase-3 activity by 15.7 and 11.8 fold (*P* < 0.001) respectively compared to cells treated with DMSO vehicle only. PB183 had little effect on caspase-3-like activity (*P* > 0.05) under these conditions, while PB28 and F281 did not activate at all the caspase-3 at these concentrations, similar to the DMSO control (Fig. 2a). To determine whether this activity was caspase-3 specific, caspase-3 was also evaluated in the presence of caspase-3 inhibitor Z-DEVD-FMK that was used as a positive control for inhibition in the experiment. The caspase-3 inhibitor Z-DEVD-FMK (1 μM) was added one hour prior to PB221 and PB282 that are the two sigma-2 ligands that showed the strongest caspase-3 activation. Five hours treatment with PB221 or PB282 caused substantial caspase activation which was completely blocked with Z-DEVD-FMK (Fig. 2b). Caspase-3 has been extensively studied as a mechanism of sigma-2 receptor ligand mediated apoptosis, and we wished to next examine the role of ROS production by our structurally diverse ligands. It turned out that following PB221 and PB282 treatment ROS production was completely blocked in the presence of α-tocopherol (Fig. 2b).

### Reactive oxygen species (ROS) involvement. Antioxidants are protective of cellular toxicity

To assess the involvement of ROS production on cell viability, we examined the effect antioxidants have on the treatment of pancreatic cancer cells with our sigma-2 ligands on cell death. The impact of antioxidants on PANC02 cell viability was assessed 24 h after treatment with PB28, PB221, PB183, F281 and PB282 (Fig. 2c). Addition of 100 μM α-tocopherol one hour prior treatment with sigma-2 ligands, rescued the viability of Panc02 cells. ROS was only decreased following sigma-2 ligands treatment in the presence of α-tocopherol. Interestingly, α-tocopherol protected against sigma-2 receptor ligands induced cell death, but N-acetyl-L-cysteine (NAC) did not, and an even higher levels of cell death was recorded when cells were co-treated with NAC and PB183 or PB282. These results were confirmed also using higher concentrations of NAC (10 mM).

### Sigma-2 ligands generate superoxide radical in the mitochondria of Panc02

**Fig. 3:**
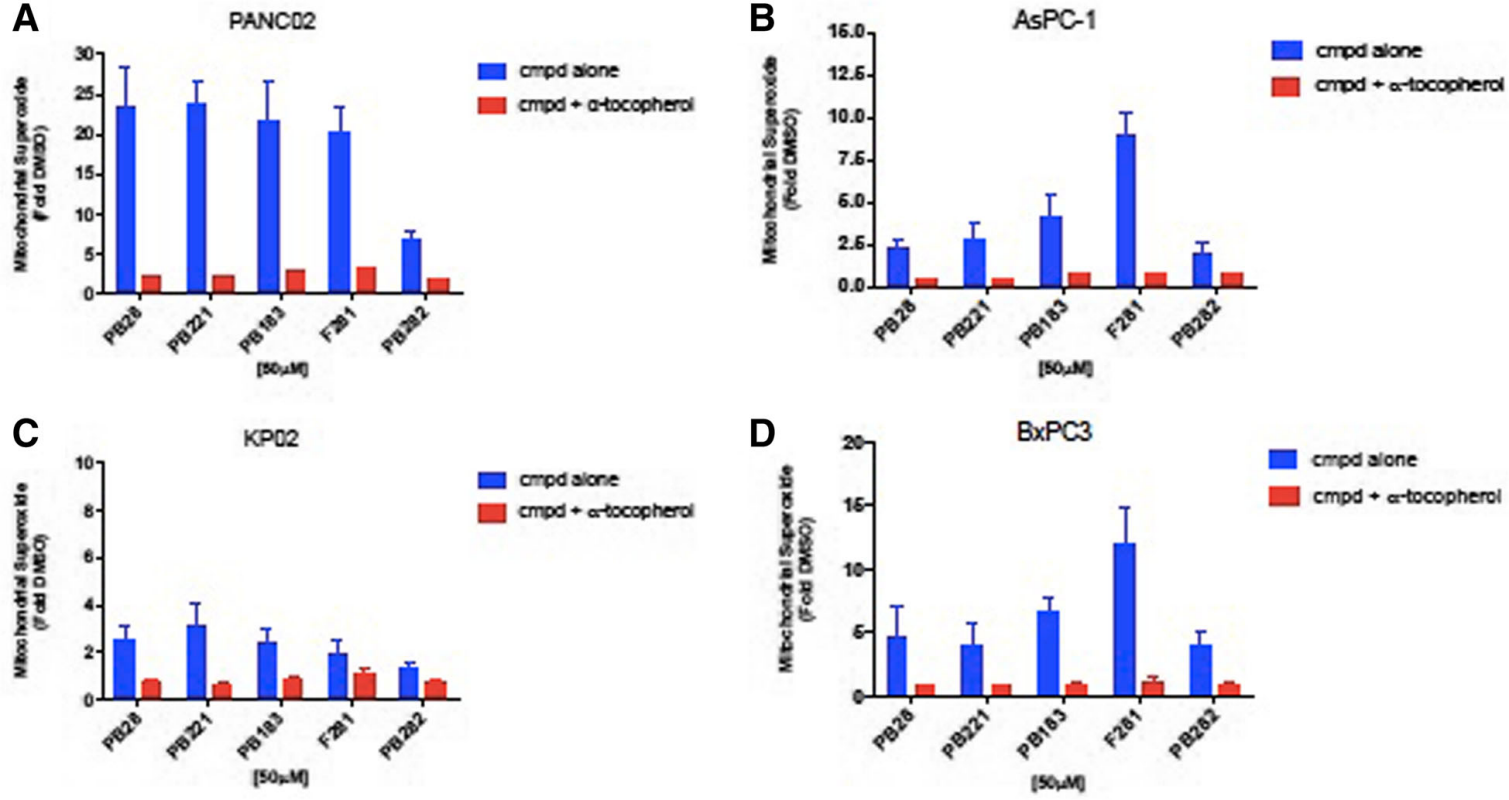
Sigma-2 Ligands generate Superoxide Radical in the Mitochondria. Mitochondrial Superoxide detection in (**a**) Panc02, (**b**) AsPC-1, (**c**) KP02, (**d**) BxPC3, after 2 h treatment with 50 μM sigma-2 ligands alone or in combination with the lipid antioxidant α-tocopherol (1 mM). Values are the means of n ≥ 3 independent experiments in triplicates with SEM, *p* < 0.0001

MitoSOX™ Red reagent is a novel fluorogenic dye specifically targeted to mitochondria in live cells. It is readily oxidized by superoxide but not by other ROS- or reactive nitrogen species (RNS)–generating systems (e.g. such as peroxides, hydroxyl radical, singlet oxygen, nitric oxide and peroxynitrite). Histograms of FACS analysis showed marked increase of mean fluorescence intensity in Panc02 cells treated with 50 μM of PB28, PB221, PB183, F281 or PB282 for 2 h (Fig. 3a). As a negative control DMSO treated cells without MitoSOX™ were used. Quantitative measurements of the mean fluorescence intensities from the samples demonstrated between 20- to 23-fold increase in MitoSOX™ fluorescence intensity with sigma-2 ligands following treatment except for cells treated with PB282 (6.7 fold compared to cells treated with DMSO). In other pancreatic cancer cell lines (AsPC1, BxPC3, KP-2) where these sigma-2 ligands do not exert cytotoxic activity, much less superoxide radical in the mitochondria was noted (Fig. 3b-d). Only F281 generated some mitochondrial superoxide in AsPC1 and BxPC3 in line with its cytotoxic effect in these cells. With the aim of obtaining a more complete picture, we evaluated the mitochondrial superoxide production in the presence of the antioxidant α-tocopherol. In all the pancreatic cancer cell lines tested for this experiment, mitochondrial superoxide production was strongly decreased following sigma-2 ligands treatment in the presence of the lipid antioxidant α-tocopherol (Fig. 3a-d).

### In vivo tumor treatment: sigma-2 ligands confer a protective effect against Panc02 tumor burden C57BL/6

**Fig. 4:**
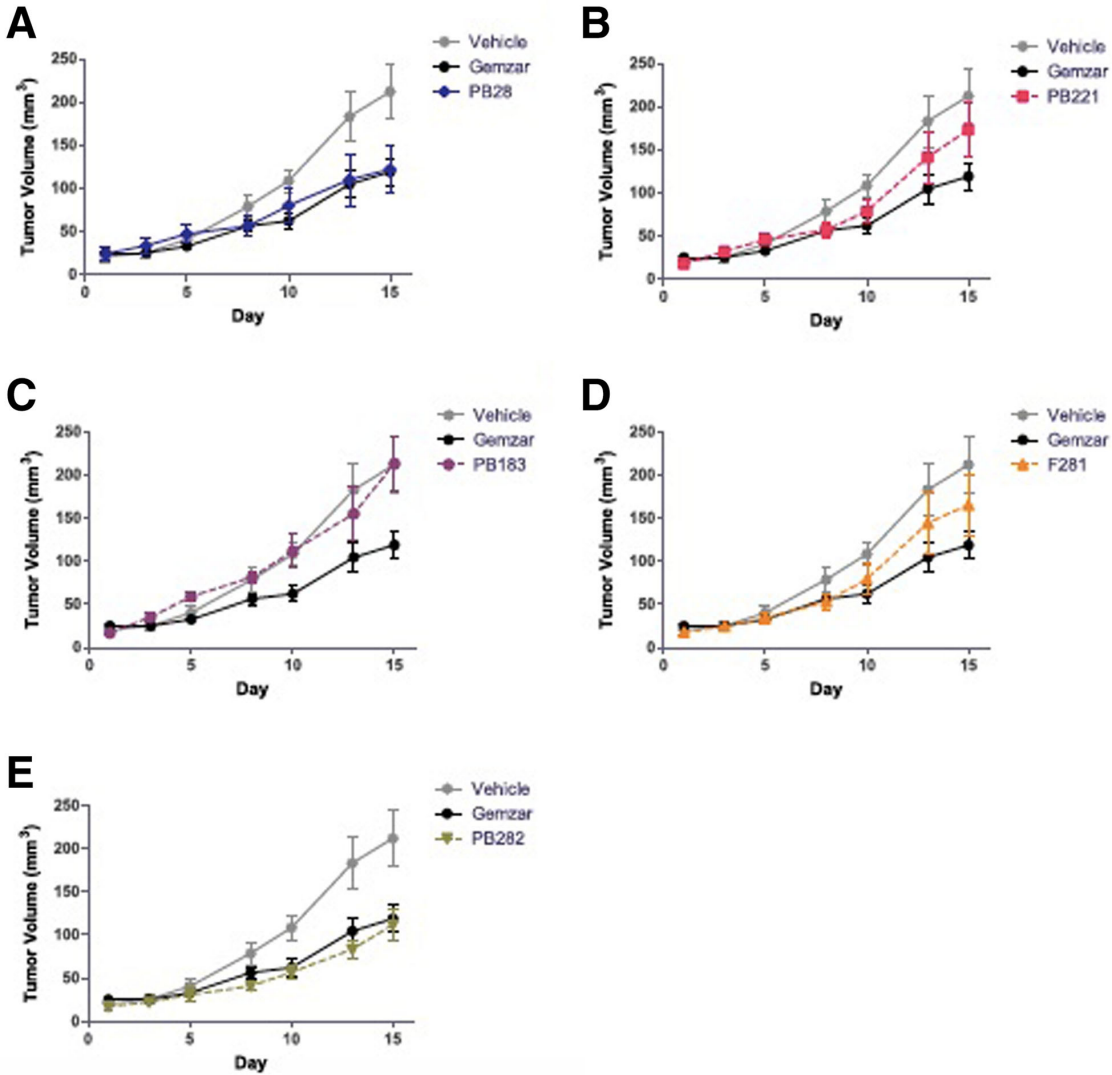
Sigma-2 ligands decrease Panc02 tumor burden in C57BL/6. One million Panc02 cells were inoculated subcutaneously into female, 10 week old C57BL/6 mice and when tumors had reached a mean diameter of 5 mm, daily sigma-2 ligand treatment or weekly gemcitabine began by i.p. injection. PB28 (**a**) and PB282 (**e**) treatment decreased tumor volume similar to gemcitabine alone. PB221 (**b**) and F281 (**d**) treatment did not confer a significantly different tumor volume than vehicle alone, while PB183 (**c**) treatment was not effective. Data represent Means ± SEM, *n* = 7-10 per group, *p* < 0.0001

**Fig. 5:**
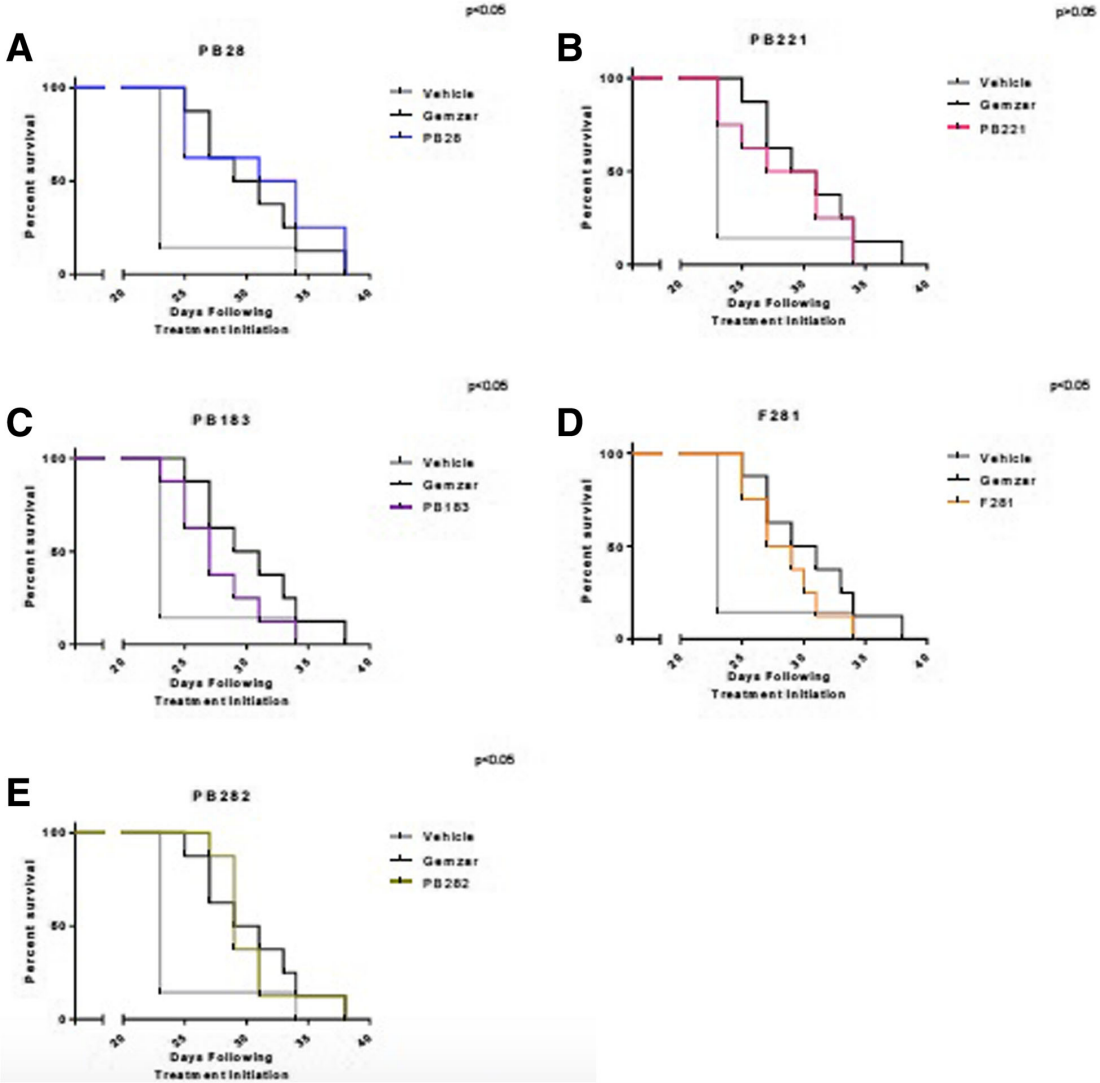
Survival Curve. Survival of mice treated with (**a**) PB28; (**b**) PB221; (**c**) PB183; (**d**) F281; (**e**) PB282 compared to the mice treated with vehicle (*p* > 0.05 for PB221 and *p* < 0.05 for all the other compounds) or Gemzar. Survival endpoints were defined as tumor diameter > 15 mm or tumor ulceration

**Fig. 6:**
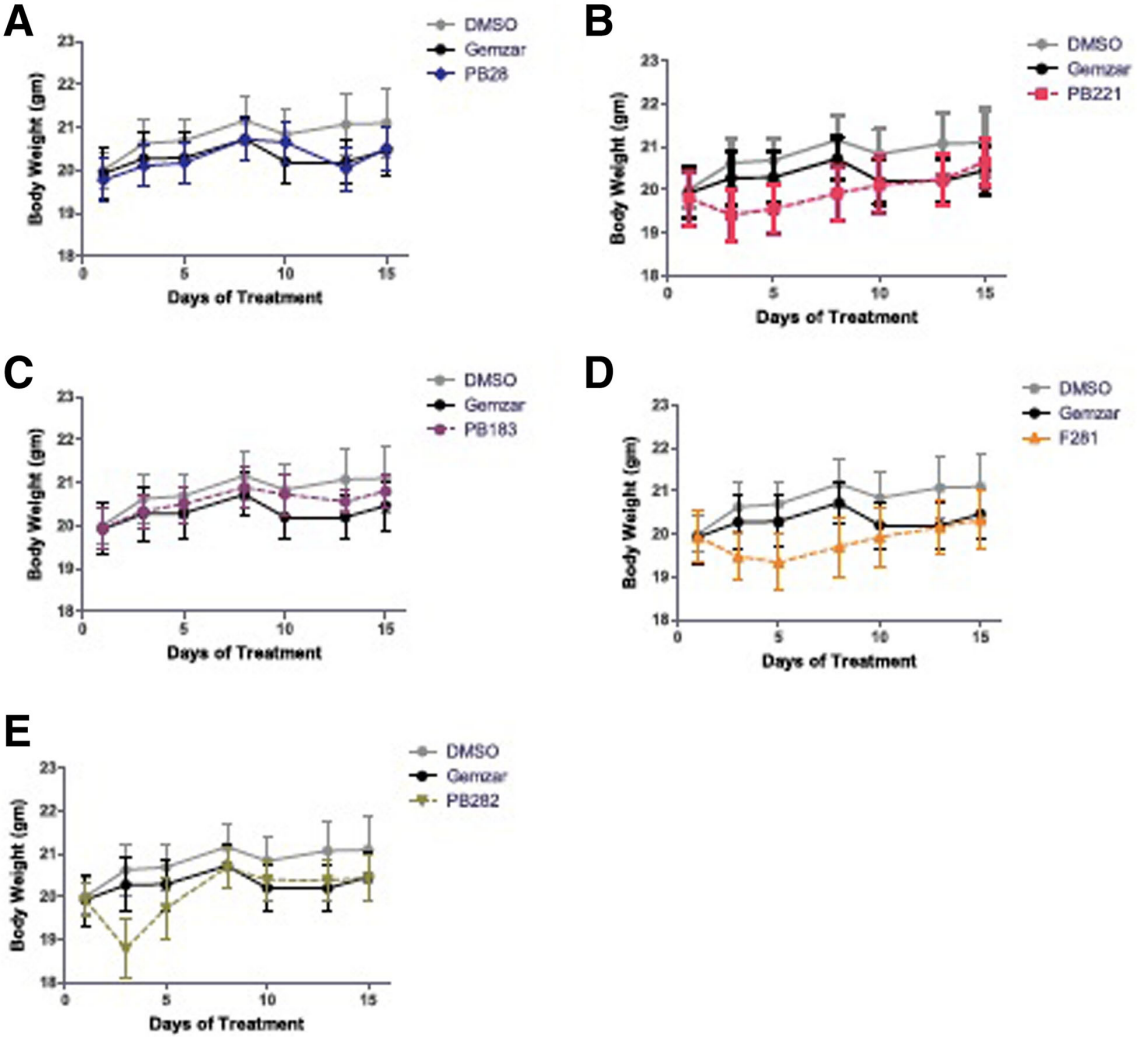
C57BL/6 mice tolerated the drugs well without signs of weight loss. **a** PB28; (**b**) PB221; (**c**) PB183; (**d**) F281; (**e**) PB282. Data represent Means ± SEM, *n* = 7–10 per group *p* < 0.0001

Multiple structurally distinct compounds (Fig. 1) with high affinity for sigma-2 receptors (Table 1) were tested for cytotoxicity against multiple pancreatic cancer cell lines in vitro (Table 1) and screened for efficacy in a mouse model of pancreatic cancer with Panc02 cells. Here, we compared the five selected sigma-2 ligands PB28, PB221, PB183, F281 and PB282 against gemcitabine used as standard control for pancreatic cancer. C57BL/6 female mice were inoculated subcutaneously with 1 × 10^6^ Panc02 cells and 10 days following tumor injection, when all mice had tumors with a diameter of approximately 5 mm, mice were randomized into treatments groups of (*n* = 7–10). Sigma-2 ligands PB28 (1.07 mg), PB221 (1.1 mg), PB183 (1.06 mg), F281 (1.05 mg), PB282 (1.09 mg), or gemcitabine (3 mg, once weekly) were given by i.p. injection daily for two weeks (Fig. 4a-e). Following conclusion of treatment, tumors were smaller for individual treatment groups PB28 (mean = 369 mm^3^), (Fig. 4a), PB282 (mean = 483 mm^3^) (Fig. 4e) and gemcitabine (mean = 395 mm^3^) compared to vehicle (mean = 924 mm^3^) (*p* < 0.0001). PB28 and PB282 treated mice had similar tumor volumes that were statistically similar to gemcitabine. Even though treatment was discontinued (on day 15), sigma-2 ligands also conferred a survival advantage for mice in this experiment. The median survival for mice treated with DMSO was 23 days compared to 27 and 29 days for mice treated with PB28 and PB282 respectively (Fig. 5a and e). All the other treatment groups had a median survival of 25 to 27 days (Fig. 5b-d). Mice appeared well throughout treatment without signs of weight loss (Fig. 6a-e) and did not experience any treatment-related deaths.

### Sigma-2 ligands have minimal toxicity in vivo

No toxicities were observed in the experiments above. However, the compounds localize to the tumors and therapeutic window might be different in non-tumor bearing animals. In order to test this hypothesis, equimolar amounts of PB28 (1.07 mg), PB221 (1.1 mg), PB183 (1.06 mg), F281 (1.05 mg), PB282 (1.09 mg) were compared in C57BL/6 naïve female mice (*n* = 3) by daily treatment for one week. In order to assess for more subtle toxicities, serum chemistries (AST, ALT, BUN, total protein, glucose, and Cr) and complete blood counts were analyzed and there were no significant differences (Table 2). Gross pathology and histology reports of brain, kidney, liver, and pancreas were not appreciably different from untreated control animals (data not shown).

**Table 2:** Sigma-2 ligands do not induce changes in blood cytology (CBC) following treatment of not tumor-bearing C57BL/6 mice

	Control	PB28	PB221	PB183	PB282	*p-*value
A.						
WBC (10^3^/μL)	10.58 ± 0.2	11.11 ± 1.97	8.12 ± 0.14	6.42 ± 1.22	5.78 ± 0.78	0.9
RBC (10^6^/μL)	7.14 ± 0.1	10.24 ± 0.3	9.59 ± 0.71	9.24 ± 0.9	9.91 ± 0.31	0.9
HGB (g/dL)	10.7 ± 0.7	15.65 ± 0.55	14.7 ± 0.8	14.85 ± 1.35	15 ± 0.4	0.4
HCT (%)	36.6 ± 0.08	53.4 ± 1.5	50.35 ± 3.75	48.8 ± 3.3	51.1 ± 1.9	0.9
MCV (fL)	51.2 ± 0.32	52.15 ± 0.05	52.5 ± 0.02	52.9 ± 1.6	51.55 ± 0.35	0.6
MCH (pg)	15 ± 0.54	15.3 ± 0.1	15.35 ± 0.35	16.1 ± 0.1	15.15 ± 0.05	0.9
MCHC (%)	29.2 ± 1.21	29.3 ± 0.2	29.25 ± 0.55	30.4 ± 0.7	29.4 ± 0.3	0.9
Platelets (10^3^/μL)	486 ± 67	643.5 ± 95.5	563 ± 17	727.5 ± 65.5	649.5 ± 167.5	0.5
B.						
BUN (mg/dL)	27 ± 0.4	32 ± 1	30.5 ± 3.5	21 ± 2	24.5 ± 0.5	0.9
Creatinine (mg/dL)	0.34 ± 0.02	0.37 ± 0.04	0.35 ± 0.03	0.37 ± 0.04	0.35 ± 0.03	0.9
ALT (μ/L)	260 ± 21	249 ± 9	219 ± 5	203 ± 34	221 ± 63	0.8
AST (μ/L)	5.6 ± 0.05	5.85 ± 0.35	5.35 ± 0.05	5.4 ± 0.5	5.5 ± 0.07	0.7
Total Protein (g/dL)	168 ± 42	194 ± 72	67.5 ± 1.5	197 ± 103	164 ± 52	0.9
Glucose (mg/dL)	62 ± 12.5	92.5 ± 22.5	64.5 ± 7.5	125 ± 55	60 ± 10	0.4

(A) Blood cytology analysis of C57BL/6 mice (*n* = 3 mice/group) treated with S2L and vehicle (control) for 5 days. The differences in complete blood count laboratory values between the two groups are not statistically significant. (B) Biochemical analysis of C57BL/6 mice (*n* = 3 mice/group) treated with sigma-2 ligands and vehicle (control) for 5 days. The differences in serum chemistries between the groups are not statistically significant

## Discussion

Currently available chemotherapeutics have poor efficacy in pancreatic cancer patients. Chemotherapeutic drugs enter normal and cancer tissues with similar kinetics, resulting in toxic effects on normal tissues often leading to changes in the therapeutic plan such as dose reductions that leads to less effective drug combinations and discontinuation of therapy. Without specific targeting of these treatments, off-site toxicities can occur and the therapeutic window can be reduced. Sigma-2 receptors ligands appear to be selective to cancers: sigma-2 receptors are highly expressed in tumor cells and allow selective targeting of cancers [38]. Sigma-2 ligands have been demonstrated to image not only pancreatic tumors in animal models [21] but, more importantly, in clinical imaging studies [39].

PB28 and PB282 compounds previously showed encouraging results in vitro and in vivo on the viability of BxPC3 pancreatic cancer cells [24]. Therefore, herein, we selected five different sigma-2 ligands, that were tested on a wide panel of human and mouse pancreatic adenocarcinoma cell lines in vitro.

Most of the compounds displayed high sigma-2 affinity and exerted appreciable cytotoxic activity in some pancreatic cancer cell lines. We showed that, in particular, F281 had a greater capacity to decrease viability in the pancreas cancer cell lines tested, except for Panc1. The mechanism by which sigma-2 ligands, at high doses, induce cell death is not completely understood. We do know that sigma-2 ligands induce apoptosis by caspase-3 dependent and independent mechanisms [16, 22, 40]. The generation of reactive oxygen species (ROS) has been well demonstrated to be both a by-product and a promoter of apoptosis and necrosis. of The apoptotic signals is intracellularly transmitted through production of ceramide or through direct effect on the mitochondria [41]. Diverse mechanisms of apoptosis by ROS are known and may be cell-type dependent, while antioxidants or intracellular enzymes such as superoxide dismutase, catalase, and glutathione peroxidase have been shown to protect against ROS. We observed that the five sigma-2 agonists induced ROS in Panc02 cells and that apoptosis could be partially ameliorated by treatment with the antioxidant α-tocopherol. On the other hand, in this study, the small diffusible hydrophilic antioxidant NAC, a precursor of glutathione, did not protect against cell death by sigma-2 ligands, resulting in an even higher toxicity in co-administration assays with PB183 and PB282. Since at low concentrations (100 μM), there are chances that NAC behaves as an oxidant (rather than acting as an antioxidant or a radical scavenger), higher concentrations of NAC (10 mM) were also used but results did not change. NAC is a general reducing agent whereas α-tocopherol protects against oxidative stress is by preventing membrane lipid peroxidation, so that we believe that our results indicate crucial differences in the intracellular sites exposed to oxidative stress by sigma-2 receptor ligands. By contrast, PB282 did not show cytotoxic activity in Panc02 cell lines. However, only PB282 and PB221, at the concentration and time point used, were able to generate a strong caspase-3 activation, which was completely blocked by α-tocopherol and by caspase-3 inhibitor Z-DEVD-FMK. Since PB28, PB183, PB221 and F281 showed cytotoxicity in Panc02 cells, these results suggest that their cytotoxic activity is caspase-3 independent, except for PB221 that strongly activated caspase-3, but both cytotoxity and caspase-3 activation involved ROS generation. In addition, all the compounds strongly increased superoxide mitochondrial radical production except for PB282. This result suggests that superoxide mitochondrial radical production is one of the factors responsible for the cytotoxic effect of these ligands. In line with this result, in other pancreatic cancer cell lines where these sigma-2 ligands do not induce appreciable cytotoxicity (AsPC1, BxPC3, KP-2), much less superoxide radical formation in the mitochondria was detected. Only F281 generated some mitochondrial superoxide in AsPC1 and BxPC3, where a moderate cytotoxicity was noticed, in line with the hypothesis that mitochondrial superoxide pathway is at least partially responsible for the cytotoxic activity caused by our compounds in pancreatic cancer cells. These data indicate that structurally diverse sigma-2 ligands can activate different pathways in a panel of diverse cancer cell lines, and for the first time mitochondrial superoxide pathway was demonstrated for sigma-2 ligands. To verify that these in vitro results could potentially translate into an anti-tumorigenic effects in vivo, we utilized the aggressive cell line Panc02 in a syngeneic model of pancreatic cancer using C57BL/6 mice. We have shown that our treatment schedule resulted in minimal off-target toxicities in vivo (measured by serum studies and body weight) and was well tolerated. Although PB282 did not show cytotoxicity in Panc02 cells in vitro, daily treatment with PB28 and PB282 produced an effect statistically similar to gemcitabine alone, while other compounds such as PB221 and F281, that showed good EC_50_ values in this cell line in vitro, did not perform as well in vivo. This could be due to metabolic effects observed in in vivo experiment. In particular, PB282 could produce active metabolites, in contrast to PB221, PB183 and F281 that are less effective in vivo despite their higher toxicity in the same tumor in vitro. We would also consider that survival would have likely been further increased if the treatment duration would have been extended since tumor volume was stabilized during the limited treatment period of only two weeks, without anticipating additional off-site toxicities. Along these lines, necropsy and laboratory evaluations demonstrated that PB28, PB221, PB183, F281 and PB282 had no major off-target effects even in naïve mice during continuous treatment as they well tolerated the drugs without signs of weight loss and no casualties as a result of drug treatments.

## Conclusions

Overall, this study confirmed sigma-2 receptors as a promising target for the targeted treatment of pancreatic tumors, while mitochondrial superoxide production was identified as a novel mechanism of sigma-2 receptor-mediated cell death.

## Abbreviations

i.p.: Intraperitoneal injection
NAC: N-acetyl-L-cysteine
RNS: Reactive nitrogen species
ROS: Reactive oxygen species
